# Smokers Show Lower Levels of Psychological Well-Being and Mindfulness than Non-Smokers

**DOI:** 10.1371/journal.pone.0135377

**Published:** 2015-08-13

**Authors:** Víviam Vargas Barros, Elisa Harumi Kozasa, Taynara Dutra Batista Formagini, Laís Helena Pereira, Telmo Mota Ronzani

**Affiliations:** 1 Department of Psychology, Human Sciences Institute, Universidade Federal de Juiz de Fora, Juiz de Fora, Minas Gerais, Brazil; 2 Department of Psychobiology, Universidade Federal de São Paulo, São Paulo, São Paulo, Brazil; 3 Instituto do Cérebro, Hospital Israelita Albert Einstein, São Paulo, São Paulo, Brazil; Georgia Regents University, UNITED STATES

## Abstract

Mindfulness is defined as “paying attention in a particular way, on purpose, in the present moment, and nonjudgmentally”. Mindfulness is associated with positive affect, life satisfaction, self-esteem, lower negative affect and rumination. Conversely, evidence suggests a relationship between nicotine dependence and psychiatric disorders. This study aimed to compare the levels of Mindfulness and Subjective Well-Being (SWB) between smokers and non-smokers. Ninety seven smokers and eighty four non-smokers participated in the study (n = 181). The Five Facet Mindfulness Questionnaire (FFMQ-BR) and the Subjective Well-Being Scale (SWBS) were used. In all the factors of SWBS, the total scores in the FFMQ-BR and in the facets of Observing and Non-Reactivity, the non-smokers scored higher than the smokers. This study suggests that smokers present lower levels of Mindfulness and SWB than non-smokers. Consequently, we propose that Mindfulness-Based Interventions (MBI) may help smokers deal with treatment and abstinence by increasing their level of SWB.

## Introduction

The concept of Mindfulness, in western psychology, is understood as a metacognitive ability, defined by Jon Kabat-Zinn as “paying attention in a particular way, on purpose, in the present moment and nonjudgmentally” [1]. Mindfulness has been defined as an intrinsic human capability that can be developed and trained, and its improvement has been connected with better health outcomes in both medicine and psychology [2]. The abilities developed by this practice allow people to reduce the negative and increase the positive affect and, consequently, improve the Subjective Well-Being (SWB) [3].

The construct of SWB has three main components; positive affect, negative affect and life satisfaction. *Positive affect* is a hedonic contentment, experienced alertness, enthusiasm and activity. *Negative affect* refers to distraction and displeasing engagement which is also transitory but includes unpleasant emotions and other distressing psychological symptoms. The dimension *life satisfaction* is a cognitive estimation; a process of judgment and general evaluation of one’s own life [4].

Studies have associated higher levels of Mindfulness with higher positive affect, life satisfaction and self-esteem, and lower negative affect and rumination [5]. On the other hand, there is evidence of a close association between nicotine dependence and mental disorders [6][7]. According to previous studies [8], the comorbidities most often associated with tobacco use are mood disorders, some anxiety disorders, and other substance use disorders.

However, there is evidence that training in Mindfulness generates an ability that acts by reducing stress, craving, withdrawal symptoms and emotional discomfort, in addition to an ability that the individual has available for the rest of his life, and can apply as a response to triggers to substance use [9]. Consequently, the objective of the current study is to compare the levels of Mindfulness and SWB between smokers and non-smokers.

## Materials and Method

### Participants

The sample was composed of individuals who participated in the validation study of the Five Facet Mindfulness Questionnaire for the Brazilian population [10]. The sample was comprised of 181 participants, out of which 97 were smokers and 84 were non-smokers. The Chi-square test was performed to verify whether the groups were similar and no significant difference was found between groups in any of the socio-demographic variables; differing only in regard to the use of tobacco. The smokers were recruited from a public health facility specialized in smoking cessation and the non-smokers were from a primary health unit and undergraduate students at the Universidade Federal de Juiz de Fora (UFJF). Table 1 shows the socio-demographic characteristics of the participants.

**Table 1:** Socio-demographic characteristics of the participants (N = 181).

		Smoker				
Demographics		Yes		No		p
		N	%	N	%	
Gender	Female	63	52.5	57	47.5	p = 0.75
	Male	34	55.7	27	44.3	p = 0.75
Schooling	Up to Junior High	40	50.6	39	49.4	p = 0.66
	High School					p = 0.66
	Complete or Incomplete	36	53.7	31	46.3	p = 0.66
	College—Complete	21	60	14	40	p = 0.66
	or Incomplete					p = 0.66
Age	Up to 44 years	32	55.2	26	44.8	p = 0.75
	45 or older	63	52.1	58	47.9	p = 0.75
Family Income in Minimum Wages^a^	Up to 3	87	52.4	79	47.6	p = 0.41
	3 or more	10	66.7	5	33.3	p = 0.41

^a^One minimum wage in Brazil is equivalent to approximately US$ 1,376.34 per month.

### Instruments

1. Socio-demographic questionnaire: used to determine the characteristics of the participants in relation to the variables; age, gender, schooling and family income.
2. Fagerström Test for Nicotine Dependence: used to inform the level of nicotine dependence among the smokers. It is a self-report questionnaire, consisting of six questions and evaluates the level of nicotine dependence (α = 0,642). The level of dependence may be evaluated through the following scores; 0–2 = very low; 3–4 = low; 5 = average; 6–7 = high and 8–10 = very high [11].
3. Five Facet Mindfulness Questionnaire (FFMQ-BR): evaluates the level of Mindfulness. It consists of 39 items that, in the Brazilian version, are divided into seven components. The names of the facets, the Cronbach’s alpha and a sample item of each facet are provided below: (1) Non-judging (α = 0,78), Item 17: “I make judgments about whether my thoughts are good or bad”, (2) Acting with awareness (automatic pilot) (α = 0,79), Item 34: “I do jobs or tasks automatically without being aware of what I’m doing”, (3) Observing (α = 0,76), Item 15: “I pay attention to sensations such as the wind in my hair or sun on my face”, (4) Describing (α = 0,76), Item 2: “I’m good at finding words to describe my feelings”, (5) Describing (items with negative formulation) (α = 0,75), Item 22: “When I have a sensation in my body, it’s difficult for me to describe it because I can’t find the right words”, (6) Non-reactivity (α = 0,68), Item 29: “When I have distressing thoughts or images I am able just to notice them without reacting”, (7) Acting with awareness (distraction) (α = 0,63), Item 5: “When I do things, my mind wanders off and I’m easily distracted” [12][10]. The Cronbach’s alpha of the total scale was 0.81.
4. Subjective Well-Being Scale (SWBS): consists of 62 items. In the first part of the scale, items 1 to 47, the items are divided into positive and negative affects (α = 0.95). High scores in the positive and negative affects suggest a high level of positive affect and a low level of negative affect, respectively. The second part of the scale, items 48 to 62, comprises the factor *life satisfaction* (α = 0.90). Samples of items of each factor include; Item 7: Cheerful (positive affect), Item 13: Depressed (negative affect) and Item 48: “I am satisfied with my life” (life satisfaction) [4].

### Data Analysis

The data were submitted to descriptive and inferential analyses. To characterize the sample, descriptive statistical analysis was used, calculating mean, median, standard deviation and frequency for the nominal and ordinal variables.

Regarding the inferential analyses, visual inspection of the Quartile-Quartile type graphs was used, along with the Kolmogorov-Smirnov test related to the participants’ scores in the FFMQ-BR and SWBS for each group. Two-way ANOVA for independent samples was used when homoscedasticity was detected by the Levene test. In cases where-n the scores did not meet that condition, the corrected values in order to assess whether there was an interaction of sex on the differences between smokers and non-smokers concerning SWB and Mindfulness was used. To evaluate whether the individual items related to positive and negative affects could differ significantly between groups, the t test for independent samples was used. In addition, to evaluate if the levels of Mindfulness and SWB differed across the nicotine dependence levels, respectively t test or the Mann-Whitney Test was used, for normal or non-normal distribution scores. In the cases where there was not a normal distribution, the nonparametric test of Mann-Whitney Test was used to evaluate if there were differences between men who smoke or not and women who smoke or not, regarding mindfulness level and subjective well-being. A correlational analysis between the Fagerström scores with mindfulness and SWB scores was also conducted, using Pearson or Spearman, depending on the normality of data. The Effect Size correlation calculated from Cohen’s d was used to evaluate the effect size for normal distributions scores and a simplified rank-biserial correlation for non-normal distributions scores [13]. The level of significance of 95% was adopted for all the statistical tests (p<0.05).

The dataset of this research was provided to Plos One and is available upon request to the corresponding author of this article. See S1 Dataset.

### Ethics Statement

All the participants signed a Consent Form after the nature of the procedure had been fully explained to them. The Committee of Ethics in Research of the Universidade Federal de Juiz de Fora approved the study under # n° 120/2011, indicating that the procedures followed were in accordance with the standards of this Committee.

## Results

As far as nicotine dependence is concerned, 36.1% of the smokers had a low score and 64% had a high score. The gender of the participant had no association with the level of nicotine dependence or with the fact of whether the participant was a smoker with p values of 0.45 and 0.64 respectively. Neither the levels of Mindfulness nor SWB were significantly influenced by the level of nicotine dependence.

It was detected that non-smokers had higher scores in all the SWB measures, including its different factors. As for Mindfulness, the non-smokers had significantly higher scores only in three measures: total score in the FFMQ-BR, Observing and Non-reactivity (Table 2). It was also noticed that the levels of Mindfulness did not differ according to gender in both groups. For the SWB factors we found that women who smoke had a significantly lower level of positive (p = 0.03) and a higher level of negative affect (p = 0.05) when compared with men who smoke and with the non-smokers group. There was no interaction of sex on the differences of Mindfulness and SWB across the groups. In addition, there was no significant correlations between severity of dependence and Mindfulness or Subjective Well-being scores.

**Table 2:** Description of the levels of Mindfulness and SWB of the participants (N = 181).

Scales	Gender	Smoker	n	M	(SD)	Md	p	Effect Size	Variable	Gender	Smoker	n	M	(SD)	Md	p	Effect Size
Total FFMQ	female	Yes	63	120.0	(15.54)	121	0.07	-0.16		female	Yes	63	22.3	(5.37)	23	0.017	0.22
	female	No	57	126.0	(20.12)	129	0.07	-0.16		female	No	57	25.1	(6.03)	25	0.017	0.22
	male	Yes	34	125.8	(17.20)	121	0.08	-0.22	Non-reactivity (FFMQ)	male	Yes	34	22.5	(6.85)	22.5	0.114	0.20
	male	No	27	133.5	(17.01)	136	0.08	-0.22		male	No	27	25.7	(5.85)	26	0.114	0.20
	Total*	Yes	97	122.0	(16.29)	121	0.02	-0.17		Total	Yes	97	22.4	(5.89)	23	0.001	0.49
	Total*	No	84	128.4	(19.40)	130	0.02	-0.17		Total	No	84	25.3	(5.94)	25	0.001	0.49
Non-judging (FFMQ)	female	Yes	63	23.6	(7.02)	25	0.28	0.10		female	Yes	63	10.0	(3.15)	10	0.346	0.09
	female	No	57	22.2	(7.03)	23	0.28	0.10	Act with Awareness (distraction) (FFMQ)	female	No	57	10.5	(3.58)	11	0.346	0.09
	male	Yes	34	25.8	(7.11)	26.5	0.41	0.11		male	Yes	34	10.8	(3.20)	11	0.554	0.08
	male	No	27	24.2	(7.02)	24	0.41	0.11		male	No	27	11.3	(3.45)	13	0.554	0.08
	Total*	Yes	97	24.3	(7.09)	25	0.19	0.10		Total	Yes	97	10.3	(3.18)	10	0.38	0.12
	Total*	No	84	22.8	(7.05)	23	0.19	0.10		Total	No	84	10.7	(3.53)	11	0.38	0.12
Act with Awareness (automatic pilot) (FFMQ)	female	Yes	63	17.5	(3.56)	19	0.472	-0.07		female	Yes	63	2.8	(0.83)	2.86	0.005	0.26
	female	No	57	17.1	(3.40)	18	0.472	-0.07		female	No	57	3.3	(0.76)	3.33	0.005	0.26
	male	Yes	34	17.6	(2.99)	17.5	0.363	0.12	Positive affect (SWBS)	male	Yes	34	3.2	(0.73)	3.26	0.131	0.19
	male	No	27	18.2	(3.17)	19	0.363	0.12		male	No	27	3.5	(0.72)	3.71	0.131	0.19
	Total*	Yes	97	17.5	(3.35)	19	0.88	0.01		Total	Yes	97	3.0	(0.81)	3	0.001	0.55
	Total*	No	84	17.5	(3.35)	18	0.88	0.01		Total	No	84	3.4	(0.75)	3.36	0.001	0.55
Observing (FFMQ)	female	Yes	63	21.8	(6.22)	22	<0.001	-0.32		female	Yes	63	3.2	(0.94)	3.12	0.006	0.25
	female	No	57	25.9	(5.71)	26	<0.001	-0.32		female	No	57	3.6	(0.93)	3.69	0.006	0.25
	male	Yes	34	22.6	(5.80)	23	0.194	-0.16	Negative Affect (SWBS)	male	Yes	34	3.6	(0.92)	3.73	0.019	0.30
	male	No	27	24.7	(6.94)	27	0.194	-0.16		male	No	27	4.1	(0.60)	4.27	0.019	0.30
	Total*	Yes	97	22.0	(6.06)	22	<0.001	-0.28		Total	Yes	97	3.3	(0.95)	3.27	0.001	0.05
	Total*	No	84	25.5	(6.12)	26	<0.001	-0.28		Total	No	84	3.8	(0.86)	3.96	0.001	0.05
Describing (FFMQ)	female	Yes	63	14.7	(4.34)	15	0.184	0.12		female	Yes	63	3.0	(0.90)	2.87	<0.001	0.33
	female	No	57	15.9	(5.63)	15	0.184	0.12		female	No	57	3.6	(0.71)	3.67	<0.001	0.33
	male	Yes	34	16.0	(5.57)	15	0.136	0.19	Life satisfaction (SWBS)	male	Yes	34	3.3	(0.74)	3.37	0.001	0.44
	male	No	27	17.9	(4.49)	19	0.136	0.19		male	No	27	3.9	(0.68)	4.13	0.001	0.44
	Total*	Yes	97	15.2	(4.82)	15	0.07	0.26		Total	Yes	97	3.1	(0.85)	3.2	<0.001	0.75
	Total*	No	84	16.5	(5.34)	18	0.07	0.26		Total	No	84	3.7	(0.71)	3.8	<0.001	0.75
Describing (items in the negative) (FFMQ)	female	Yes	63	10.2	(3.20)	10	0.238	-0.10		female	Yes	63	3.0	(0.73)	2.92	<0.001	0.44
	female	No	57	9.4	(3.65)	9	0.239	-0.11		female	No	57	3.5	(0.67)	3.56	<0.001	0.44
	male	Yes	34	10.5	(3.74)	11.5	0.263	0.15	Total SWBS	male	Yes	34	3.3	(0.67)	3.28	0.005	0.34
	male	No	27	11.6	(3.38)	12	0.263	0.15		male	No	27	3.8	(0.54)	3.95	0.005	0.34
	Total*	Yes	97	10.3	(3.38)	10	0.68	0.05		Total	Yes	97	3.1	(0.72)	3.1	<0.001	0.70
	Total*	No	84	10.1	(3.70)	11	0.68	0.05		Total	No	84	3.6	(0.64)	3.68	<0.001	0.70

*Difference Adjusted by Gender using ANOVA Two-Way.

The individual analysis of the SWBS regarding affects showed that all the items of the negative affect were higher among smokers. The opposite was also true for non-smokers.

## Discussion

Considering the specificities of substance dependence disorder, as in the case of nicotine, it is interesting to notice a significantly higher level of Mindfulness among non-smokers in the facets Observing and Non-reactivity. Moreover, smokers presented with significantly lower levels of life satisfaction, positive affect and general SWB, and higher levels of negative affect, when compared with non-smokers. As largely described in the literature [14], such results suggest that those components may play a role on the decision to smoke or not. There were also some tentative gender differences regarding the Mindfulness categories assessed, but large discrepancies in the group sizes did not allow strong conclusions to be drawn at this point.

The items of the SWBS showed that all the items of the factor “Negative Affect” were significantly different between groups, being higher among smokers. Most of those items regard depressive or anxiety states, common in this population [15]. The item “anxious” presented the greatest difference between the groups. This finding may be related to the differences detected between groups in the facets Observing and Non-reactivity of the FFMQ-BR. Since those smokers feel anxious, they might experience difficulty observing their mental events, therefore, they would be caught in a vicious circle in which they would not stop to observe and perceive facts, and would react to them through maladaptive automatic behaviors which, in turn, could increase anxiety. As automatic reactions are often accompanied by unpleasant consequences, smokers would evaluate their life more negatively and less positively.

In a study with heavy drinkers, the authors observed that the association between craving and heavy alcohol use was weaker among those who had received training in Mindfulness [16]. These findings indicate that Mindfulness could weaken the relationship between automatic mental processes and substance use. Still in line with the above findings, previous studies observed that women who presented average and low levels of Mindfulness in the facets “Non-Reactivity” and “Describing” smoked more often, while the women with higher levels of Mindfulness presented a lower frequency of use [17].

Furthermore, regarding the relationship between Mindfulness and SWB, the authors evaluated participants from the general population with moderate or severe psychological problems [3]. It was asserted that the individuals who underwent a Mindfulness-Based Cognitive Therapy presented better attention regulation, with a focus on the present, which could have increased positive affect. Additionally, different attitudes during daily activities, with greater nonjudgmental acceptance of the situations, were significantly related to a reduction in the negative affect. Given these results, it is possible to infer that the changes resulting from the intervention are associated with an increase in SWB and life satisfaction, since the experience of and focus on negative episodes do not overlap the positive aspects.

Some recent studies have already corroborated the effectiveness of Mindfulness-based interventions for smoking, and suggest that adherence to meditation practices is positively correlated to smoking cessation and reduction of stress and distress [9][18]. It is proposed, therefore, that Mindfulness-based interventions used as adjunct therapy could significantly increase the success of the interventions.

The authors found that the guidance of Mindfulness helped patients face smoking triggers through the reduction of negative affects and depression, having an impact on the level of nicotine dependence as well [19]. It helped individuals re-perceive their experiences and find the adequate response to triggers, as opposed to the behavioral strategy of suppressing thoughts, which does not allow conscious analysis of thoughts and, in turn, makes it difficult to change responses to triggers. It is widely recognized, from a clinical standpoint, that the mood and the motivation of the patients strongly influence their adherence to treatment, in addition to influencing their behavior and the self-efficacy in the face of craving, withdrawal symptoms or exposure to triggers to use the substance. Similarly, there is evidence that specific personality traits such as negative affect are strong predictors of failure during attempts to quit smoking [20].

Mindfulness-based practices may be an alternative for those who need a more intensive approach and do not adapt to self-help groups [21]. Previous studies also show that the level of Mindfulness might increase further, even after the end of the intervention, demonstrating that participants are able to learn the practices and insert them into their daily routine [5]. We propose, therefore, that MBI used as an adjunct therapy could increase the success of the treatment.

The study has some limitations, however. Due to its cross-sectional design, it was not possible to determine the causal direction of the findings, making it difficult to draw more precise conclusions about the mutual influence between Mindfulness and SWB, and their relation to tobacco smoking. This study was based on self report questionnaires and the answers may have been affected by social desirability or by the cognitive affect of nicotine on the system of the respondents, as the time since last smoking before answering the questionnaires was not controlled. In addition, it is not possible to generalize the results to all smokers worldwide, since the study included only Brazilian smokers that may have regional characteristics not extendable to other countries. Future studies should also consider gender differences.

Even though our results are preliminary, they suggest that tobacco smokers, especially women, present lower levels of Mindfulness and SWB than non-smokers. Consequently, we propose that Mindfulness-based Interventions (MBI) may help smokers to deal with the treatment and abstinence by increasing their level of Subjective Well-Being.

## Supporting Information

S1 Dataset The dataset of this research was provided to Plos One and is available upon request to the corresponding author of this article. (SAV)
